# Deciphering the expression patterns of homologous recombination-related lncRNAs identifies new molecular subtypes and emerging therapeutic opportunities in epithelial ovarian cancer

**DOI:** 10.3389/fgene.2022.901424

**Published:** 2022-09-29

**Authors:** Tian Hua, Xiao-Chong Zhang, Wei Wang, Yun-Jie Tian, Shu-Bo Chen

**Affiliations:** ^1^ Department of Gynecology, Affiliated Xingtai People Hospital of Hebei Medical University, Xingtai, China; ^2^ Department of Oncology, Affiliated Xingtai People Hospital of Hebei Medical University, Xingtai, China; ^3^ Department of Obstetrics and Gynecology, Second Hospital of Hebei Medical University, Shijiazhuang, China; ^4^ Department of Obstetrics and Gynecology, Fourth Hospital of Hebei Medical University, Shijiazhuang, China

**Keywords:** homologous recombination-related lncRNAs in ovarian cancer, homologous recombination deficiency, lncRNA, prognosis model, cluster, epithelial ovarian cancer

## Abstract

Epithelial ovarian cancer (EOC) is the leading killer among women with gynecologic malignancies. Homologous recombination deficiency (HRD) has attracted increasing attention due to its significant implication in the prediction of prognosis and response to treatments. In addition to the germline and somatic mutations of homologous recombination (HR) repair genes, to widely and deeply understand the molecular characteristics of HRD, we sought to screen the long non-coding RNAs (lncRNAs) with regard to HR repair genes and to establish a prognostic risk model for EOC. Herein, we retrieved the transcriptome data from the Genotype-Tissue Expression Project (GTEx) and The Cancer Genome Atlas (TCGA) databases. HR-related lncRNAs (HRRlncRNAs) associated with prognosis were identified by co-expression and univariate Cox regression analyses. The least absolute shrinkage and selection operator (LASSO) and multivariate stepwise Cox regression were performed to construct an HRRlncRNA risk model containing AC138904.1, AP001001.1, AL603832.1, AC138932.1, and AC040169.1. Next, Kaplan−Meier analysis, time-dependent receiver operating characteristics (ROC), nomogram, calibration, and DCA curves were made to verify and evaluate the model. Gene set enrichment analysis (GSEA), immune analysis, and prediction of the half-maximal inhibitory concentration (IC_50_) in the risk groups were also analyzed. The calibration plots showed a good concordance with the prognosis prediction. ROCs of 1-, 3-, and 5-year survival confirmed the well-predictive efficacy of this model in EOC. The risk score was used to divide the patients into high-risk and low-risk subgroups. The low-risk group patients tended to exhibit a lower immune infiltration status and a higher HRD score. Furthermore, consensus clustering analysis was employed to divide patients with EOC into three clusters based on the expression of the five HRRlncRNAs, which exhibited a significant difference in checkpoints’ expression levels and the tumor microenvironment (TME) status. Taken together, the results of this project supported that the five HRRlncRNA models might function as a biomarker and prognostic indicator with respect to predicting the PARP inhibitor and immune treatment in EOC.

## Introduction

Epithelial ovarian cancer (EOC) is the third most common gynecological carcinoma among females in the world (Momenimovahed et al., 2019). In 2015, it was estimated that there were 52,100 new cases and 22,500 cancer-related deaths in China (Chen et al., 2016). Unfortunately, the non-specific symptoms are hard to recognize and may be confused with other gastrointestinal tract diseases, which usually results in delays in diagnosis and treatment for most patients with EOC. In advanced stages, primary cytoreductive surgery, followed by platinum-based chemotherapy, remains the standard treatment for EOC (Kim et al., 2012). Although complete remission is generally reached during the initial stage of treatment, approximately 70% of the patients will recur within 24 months, and what follows is the rapid emergence of resistance to chemotherapy (Elke et al., 2017). The 5-year overall survival (OS) of patients is around 30%–40%, with no major breakthroughs for several decades (Allemani et al., 2015). There is increasing evidence that heterogeneity in morphology and the molecular level is the key characteristic of EOC (Blagden, 2015). More understanding of the molecular characterization of EOC may serve the purpose of earlier detection, prediction of clinical prognosis, and individual therapy selection in patients with refractory or recurrent disease. Previous molecular studies have revealed that a wide range of genomic variability was associated with different histological subtypes in EOC (Kurman and Shih, 2011). For instance, the analysis from The Cancer Genome Atlas (TCGA) research network stratified EOC into four promoter methylation subtypes, a transcriptional signature associated with survival duration based on the profile of RNA and microRNA expression (Cancer Genome Atlas Research Network, 2011). However, there is still a need to develop more molecular stratification methods for the accurate prediction of chemotherapy resistance and response to target drugs in EOC.

Homologous recombination (HR) repair is an important pathway that contributes to the double-stranded DNA break repair. Germline mutations of BRCA1 and BRCA2 are the main mechanisms involved in homologous recombination deficiency (HRD). Moreover, other mechanisms, such as germline and/or somatic mutations in the other HR repair genes and epigenetic modifications, generate a characteristic mutational signature (Guo et al., 2018; Alexandrov et al., 2020; Guo et al., 2021) and also play a certain role in HRD. It is well-known that patients with HRD exhibit specific clinical features and excellent responses to platinum-based chemotherapy and poly (ADP-ribose) polymerase (PARP) inhibitors. Hence, the HRD status in EOC tumor cells acts as a potential predictor of the response to PARP inhibitors in clinical practice based on several blockbuster clinical trials (Garrido et al., 2021). Even if the tumor is sporadic, the identification of an HRD phenotype is still conducive to choosing personalized therapy (Rojas et al., 2016). An increasing body of evidence indicated that long non-coding RNAs (lncRNAs) have an effect on the regulation of chromosomal remodeling, DNA methylation, histone modification, and genomic imprinting, as well as RNA metabolism (Wang and Chang, 2011). The aberrant expression of lncRNA is demonstrated in various types of cancer, involving important biological processes such as tumorigenesis, apoptosis, invasion and metastasis, and metabolism (Jiang et al., 2016; Li et al., 2020a). Numerous lncRNAs have been shown to participate in DNA repair pathways. For example, in prostate cancer, PCAT-1 was proved to have a prominent inhibitory effect on the HR activity by a direct interaction with the 3′UTR of BRCA2, thus affecting subsequent post-transcriptional suppression of BRCA2 (Prensner et al., 2014). TODRA, another famous HRR pathway-regulating lncRNA, indicates the promotion of HR efficiency in a RAD51-dependent manner through dual regulation of RAD51 expression and activity (Gazy et al., 2015). However, there is still a lack of systematic analysis for identifying the distribution of HR-associated lncRNAs and their correlation with clinical characteristics in EOC. Thus, it is necessary to conduct a risk model based on the HR-related lncRNA (HRRlncRNA) and to establish subtypes for personalized treatments and prognosis prediction.

In this study, we first identified an HRRlncRNA risk model in EOC by analyzing the public data downloaded from TCGA and GTEx databases. Second, the biological role of the risk model was investigated with regard to the correlation with clinical features, patients’ prognosis, and immune infiltration level, as well as drug sensitivity. Furthermore, consensus clustering analysis was conducted to identify EOC subtypes based on the HRRlncRNAs and to determine the relationships between the clusters with clinical characteristics.

## Materials and methods

### Epithelial ovarian cancer study data input

The public RNA-seq level 3 sequencing data and corresponding clinical information on 379 EOC cases were obtained from the UCSC Xena database https://xenabrowser.net/(Goldman et al., 2020); in addition, 88 normal human ovarian cases of RNA-seq level 3 sequencing data were downloaded from the UCSC Xena database related to Genotype-Tissue Expression Project (GTEx) (https://xenabrowser.net) as well. BRCA1 and BRCA2 mutations were downloaded from the GDC portal (https://portal.gdc.cancer.gov/).

### Development of the prognostic predictive signature

The 15 HR-related genes (HRRGs) were selected based on clinical evidence of sensitivity to PARP inhibitors and immunotherapy (Wang et al., 2021; Zhao et al., 2021). All human lncRNAs were selected by biotypes related to lncRNAs with the file gtf of GRCh38 obtained from the NCBI. To screen out HRRlncRNAs, according to the expression of HRRGs and lncRNAs, the Pearson correlation was utilized to assess the correlation, and we obtained 402 HRRlncRNAs (correlation coefficient >0.3 and *p*-value < 0.001). Then, the Wilcoxon test between TCGA and GTEx database cases was employed to get 206 differentially expressed lncRNAs [|log2FC| > 1 and false discovery rate (FDR) < 0.05]. To build a robust model, we separated the entire dataset into training and testing datasets at a ratio of 1:1; a univariate Cox regression analysis was conducted to obtain 29 HRRlncRNAs related to survival (R package: “survival”). Then, the least absolute shrinkage and selection operator (LASSO) Cox regression and multivariate stepwise Cox regression (R packages: “glmnet” and “survival”) were conducted, and we acquired a five-HRRlncRNA risk model in the final step. The EOC cases were split into low- and high-risk groups by the median value. We used a time-dependent receiver operating characteristic (ROC) curve to assess the predictive value of the prognostic gene signature for overall survival (R package: “timeROC”).

### Development of nomogram, calibration, and decision curve analysis

Multivariate Cox regression analysis of clinical features (age, stage, grade, and tumor residual) and the risk score were included to build the nomogram (R package: “rms”), and the 1-, 3-, and 5-year calibration curves were generated to confirm the accuracy of the nomogram. In addition, a decision curve analysis (DCA) curve was assessed to choose the prime model that has the best clinical net benefit (R package: “ggDCA”).

### Functional enrichment analysis of gene set enrichment analysis

GSEA software (v4.2.3) from MSigDB (http://www.gsea-msigdb.org/gsea/downloads.jsp) (Mootha et al., 2003; Subramanian et al., 2005) was applied to screen the significantly enriched GO and KEGG pathways between the high-risk and low-risk groups with the metrics of *p* < 0.05 and FDR <0.25.

### Immune infiltration analysis

The ESTIMATE algorithm (R package: “ESTIMATE”) was conducted to derive scores from the StromalScore, ImmuneScore, and ESTIMATEScore. A single-sample gene set enrichment analysis (ssGSEA) was employed to quantify immune cells and immune function (R packages: “GSVA” and “GSEABase”). With a comprehensive assessment of the immune infiltration state website TIMER2.0 (Li et al., 2020b) (http://timer.cistrome.org/), we downloaded a calculated file (TIMER, CIBERSORT, CIBERSORT-ABS, quanTIseq, MCPcounter, xCell, and EPIC methods included). We also assessed the relevancy between the risk score and immune infiltration score using Spearman’s correlation analysis.

### Homologous recombination deficiency score source and drug sensitivity assessment

We obtained HRD scores from the previous study (Knijnenburg et al., 2018). The half-maximal inhibitory concentration (IC_50_) of chemotherapy drugs from the Genomics of Drug Sensitivity in Cancer (GDSC) database (https://www.cancerrxgene.org/) (Geeleher et al., 2014) was retrieved to analyze that of chemotherapy drugs in TCGA dataset (R package: “pRRophetic”).

### Determination of molecular subtypes based on the risk score by consensus clustering

ConsensusClusterPlus extends the consensus clustering algorithm (including item tracking, item-consensus, and cluster-consensus plots); we used a consensus matrix and CDF plot to determine the best cluster number of subtypes (Wilkerson and Hayes, 2010) and obtained three clusters. T-distributed stochastic neighbor embedding (t-SNE) and principal component analysis (PCA) were completed by the Rtsne R package.

### Statistical analysis

We used R software (version 4.1.1) to conduct all statistical analyses. A *p*-value of not more than 0.05 was considered statistically significant unless noted otherwise; the *p*-value was notated as follows: * if *p*-value < 0.05, ** if *p*-value < 0.01, and *** if *p*-value < 0.001. The log-rank test was used to test the null hypothesis that there is no difference between the populations in the probability of an event (here a death) at any time point; the Kruskal–Wallis test by rank is a non-parametric alternative to the one-way ANOVA test, which extends the two-sample Wilcoxon test in the situation where there are more than two groups.

## Results

**FIGURE 1:**
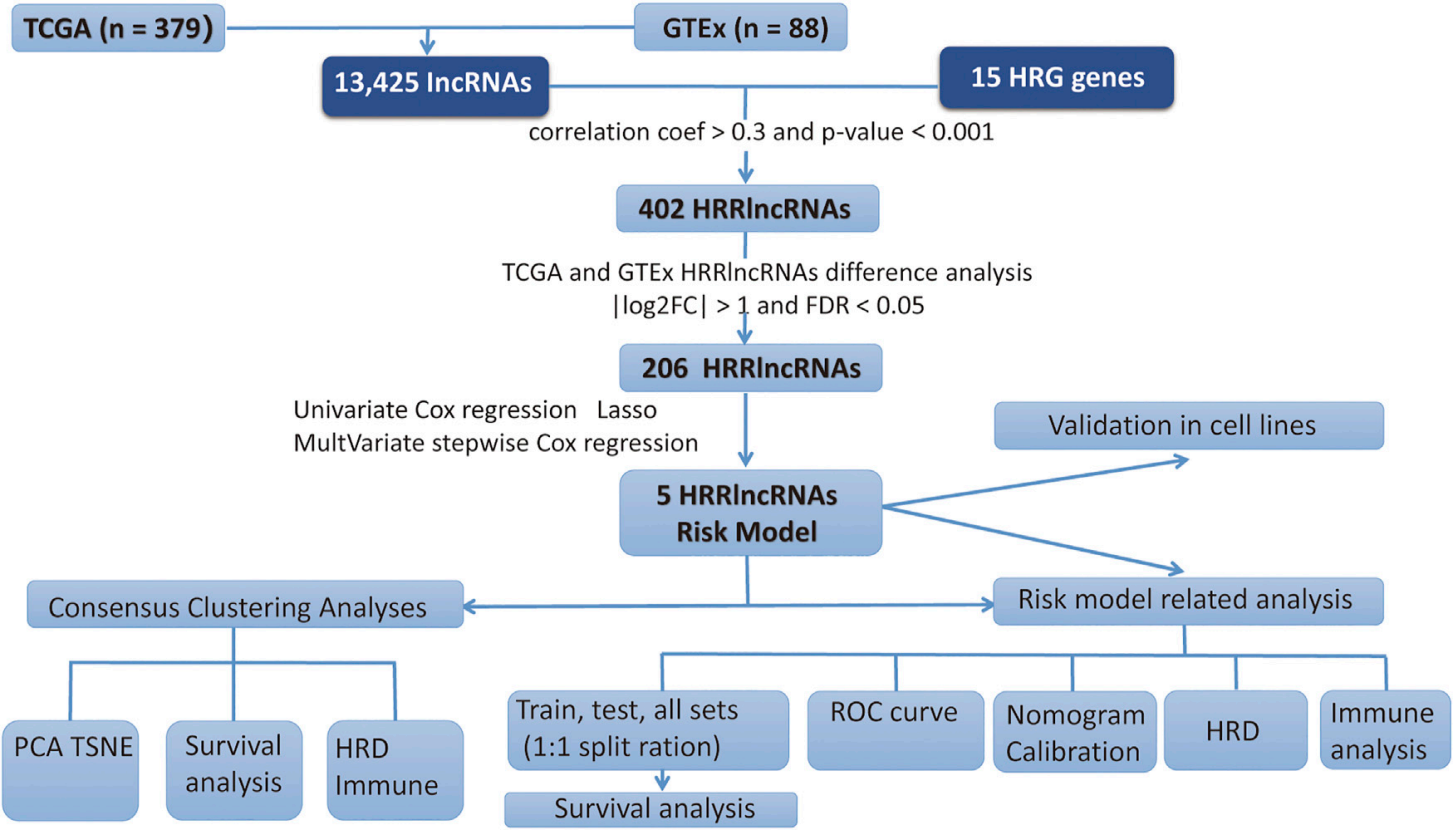
Workflow chart of this study.

In the present study, we gathered data from 379 EOC tumor and 88 normal cases from TCGA and GTEx databases. The workflow chart of the study is displayed in Figure 1.

### HRRlncRNAs in epithelial ovarian cancer

**FIGURE 2:**
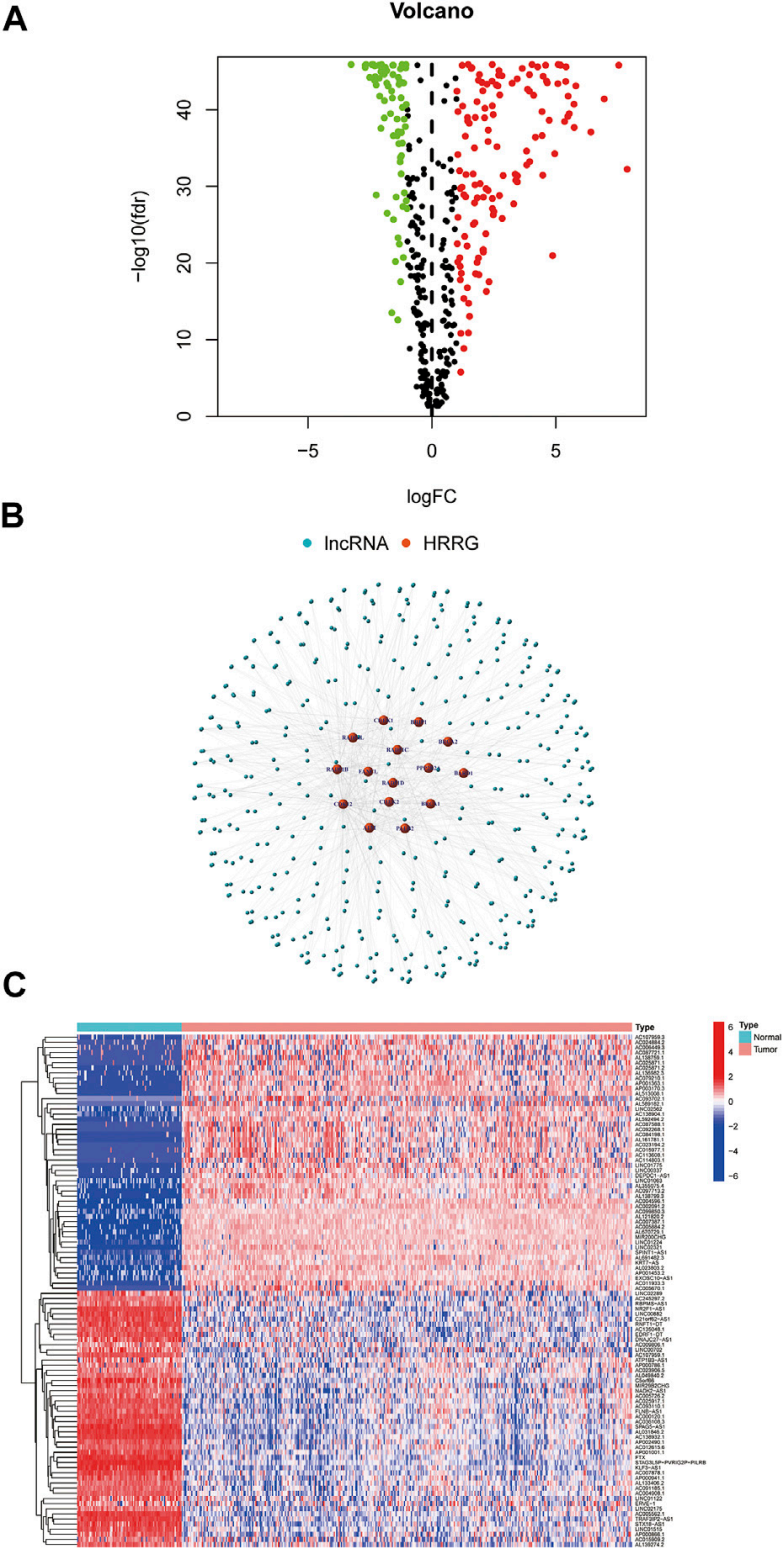
Determination of homologous recombination (HR)-related lncRNAs (HRRlncRNAs) in patients with epithelial ovarian cancer (EOC). **(A)** Volcano plot of the differentially expressed HR-related genes (HRRGs). **(B)** Network between HRRGs and lncRNAs (correlation coefficients >0.3 and *p* < 0.001). **(C)** Top 50 differentially expressed HRRlncRNAs were sorted by Log2 fold change between tumor and normal tissues.

On account of correlation coefficients (correlation coefficients >0.3 and *p* < 0.001) of 15 HRRGs and differentially expressed lncRNAs (|Log2FC| > 1 and *p* < 0.05) between TCGA and GTEx patient samples, we finally obtained 206 HRRlncRNAs, of which 132 HRRlncRNAs were upregulated and the rest 74 were downregulated (Figure 2A). The network relationship between HRRGs and lncRNAs is plotted in Figure 2B, and the detailed data are given in Supplementary Table S1. The top 50 differentially expressed HRRlncRNAs of TCGA and GTEx patient samples are shown in Figure 2C, and all differentially expressed HRRlncRNAs are supplied in Supplementary Table S2, where clear expression trends could be seen.

### HRRlncRNA risk model construction and validation in epithelial ovarian cancer

**FIGURE 3:**
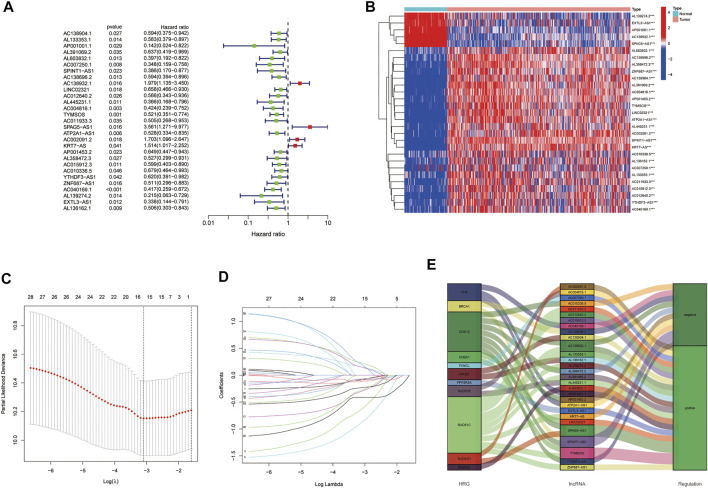
Establishment of the homologous recombination (HR)-related lncRNA (HRRlncRNA) prognostic model in epithelial ovarian cancer (EOC). **(A)** Prognosis-associated lncRNAs were extracted by univariate Cox regression analysis. **(B)** Expression profiles of 29 prognostic HRRlncRNAs. **(C)** Ten-fold cross-validation for variable selection in LASSO regression analysis. **(D)** LASSO coefficient profile of five HRRlncRNAs. **(E)** Sankey diagram of HRRGs and HRRlncRNAs.

Among the 206 differentially expressed HRRlncRNAs, 29 HRRlncRNAs were found to be significantly correlated with overall survival (OS) of EOC patients based on univariate Cox regression analysis (*p* < 0.05, Figure 3A, Supplementary Table S3), and the heatmap of 29 HRRlncRNA differential expression is shown in Figure 3B, which illustrates clear expression trends. To prevent overfitting the prognostic signature, we adopted the Lasso Cox regression on the 29 HRRlncRNAs, according to the optimum lambda value; furthermore, a multivariant stepwise Cox regression was conducted. A total of five HRRlncRNAs were finally exacted to be related to homologous recombination in EOC construction (Figures 3C,D). Additionally, the Sankey diagram was adopted to display the relationships of genes and lncRNAs with their risk types (Figure 3E). Subsequently, the risk score was calculated with the following formula:

Risk score = AC138904.1 * (-0.5307) + AP001001.1 * (-1.6239) + AL603832.1 * (-1.3534) + AC138932.1 * (0.8097) + AC040169.1 * (-0.8363).

**FIGURE 4:**
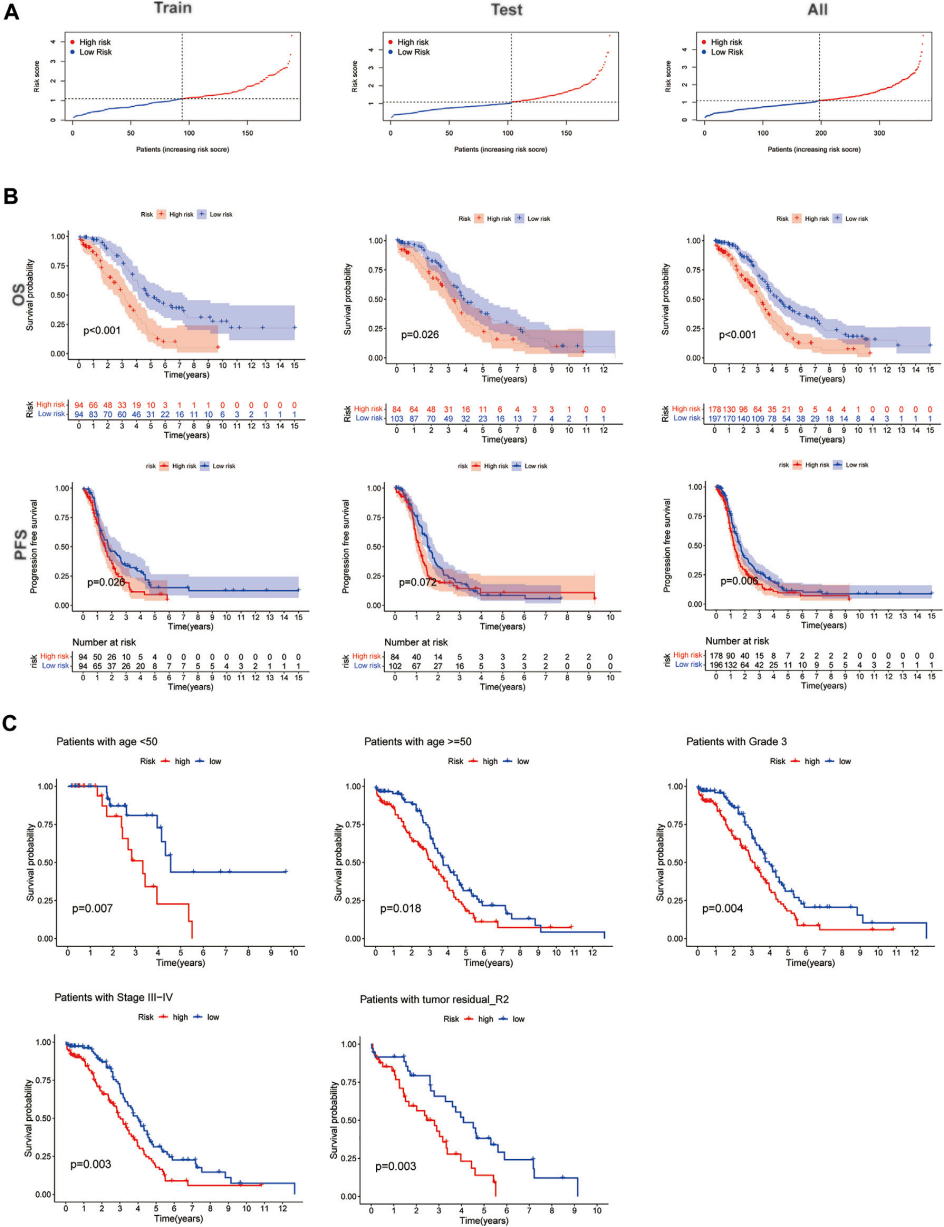
Prognosis value of the five homologous recombination (HR)-related lncRNA (HRRlncRNA) models in the training, testing, and all sets. **(A)** Presenting the HRRlncRNA model based on the risk score of the training, testing, and all sets, respectively. **(B)** Survival time and survival status between high- and low-risk subgroups in the training, testing, and all sets, respectively. Overall survival, OS; progression-free survival, PFS. **(C)** Kaplan−Meier survival curves of the overall survival (OS) prognostic value stratified by the age, grade, stage, and tumor residual size between high- and low-risk subgroups in the entire set.

The risk score median value was used as a cutoff to divide 379 EOC cases into the high- and low-risk groups. Risk score distribution was illustrated in the training, testing, and entire datasets, respectively. The risk score distribution is presented in Figure 4A; the Kaplan−Meier curve revealed that EOC patients with low-risk scores had a better OS probability than those with high-risk scores; the progression-free survival (PFS) was also significant in training and entire sets but not in the testing set (Figure 4B). Additionally, when we involved the conventional clinicopathological features, such as age, grade, stage, and tumor residual size factors, the result also agreed with the aforementioned conclusion (Figure 4C).

### Independent prognostic value of the risk model

**FIGURE 5:**
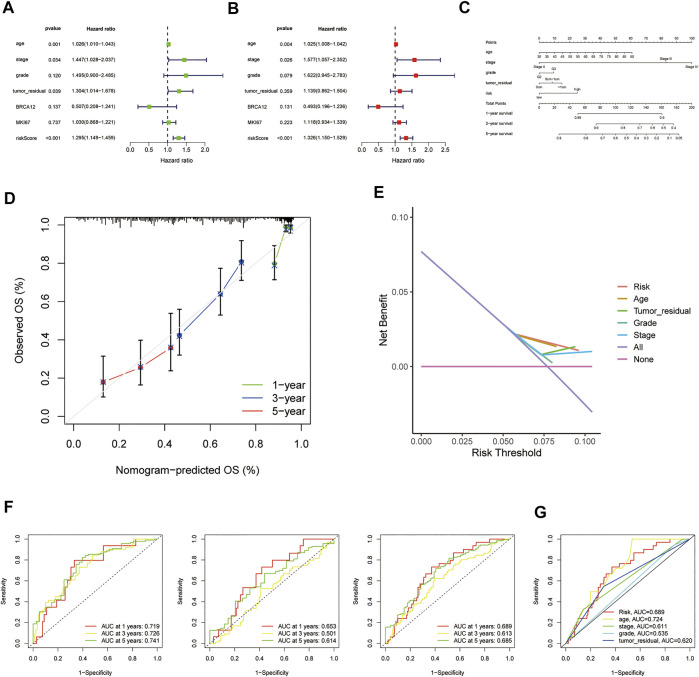
Nomogram and assessment of the risk model based on five homologous recombination (HR)-related lncRNAs (HRRlncRNAs). **(A,B)** Univariate Cox and multivariate regression analyses of the risk score and clinical factors with overall survival (OS). **(C)** Nomogram that integrated the risk score, age, grade, stage, and tumor residual size predicted the probability of the 1-, 3-, and 5-year OS. **(D)** Calibration curves for 1-, 3-, and 5-year OS. **(E)** Decision curve analysis (DCA) of the nomogram in TCGA cohort for evaluating the clinical usefulness in 1-year OS. **(F)** 1-, 3-, and 5-year time-dependent receiver operating characteristic (ROC) curves of the training, testing, and entire sets, respectively. **(G)** ROC curves of the risk score, nomogram total score, and clinical characteristics.

In consideration of the contributions of other characteristics to patient prognosis, a nomogram was made to forecast the survival risk in EOC patients. At first, the univariate and multivariate Cox regression analyses were employed to decide the independent prognostic factors of the risk score from the five HRRlncRNAs, namely, age, disease stage, histological grade, tumor residual size, BRCA1/2 mutations, and MKI67 in EOC; the results demonstrated that the risk score of the model was the independent prognostic factor for EOC patients (*p* < 0.001, HR = 1.295, and 95% CI = 1.149–1.459; Figure 5A; and *p* < 0.001, HR = 1.326, and 95% CI = 1.150–1.529; Figure 5B). As revealed by univariate analysis, age (*p* = 0.001), stage (*p* = 0.034), and tumor residual size (*p* = 0.039) in predicted dismal OS and another two independent prognostic factors, age (*p* = 0.018, HR = 1.021, 95%CI = 1.003–1.038, Figure 5B) and stage (*p* = 0.018, HR = 1.611, 95%CI = 1.086–2.390, Figure 5B), were derived by the future multivariate Cox regression. Moreover, a nomogram was made to predict 1-, 3-, and 5-year OS incidences of EOC patients (Figure 5C), and the 1-, 3-, and 5-year calibration plots were used to verify that the nomogram had a good concordance with the prediction of 1-, 3-, and 5-year OS (Figure 5D). We conducted DCA analysis to assess the clinical practicalities of the nomogram by quantifying the net benefits against a range of threshold probabilities, and the results demonstrated the prognosis prediction based on the HRRlncRNA risk model could add more net benefit than treating either none or all patients (Figure 5E). In order to evaluate the sensitivity and specificity of the risk model on the prognosis, ROC was performed. We also illustrated the outcomes of ROC with the area under the ROC curve (AUC). The HRRlncRNA risk models all displayed fine AUC values (at 1, 3, and 5 years) in ROC analysis in the training set, testing set, and all sets individually (Figure 5F), revealing effective prediction of survival by the HRRlncRNA risk signature. In terms of area under the ROC curve (AUC), the 1-, 3-, and 5-year AUCs of the training dataset were 0.719, 0.726, and 0.741, those of the testing dataset were 0.653, 0.501, and 0.614, and those of the entire dataset were 0.689, 0.613, and 0.685, respectively (Figure 5F). Of the 3-year ROC of the risk model, the risk score (0.689) displayed its predominant predictive ability (Figure 5G).

### Functional analysis based on the risk score signature

**FIGURE 6:**
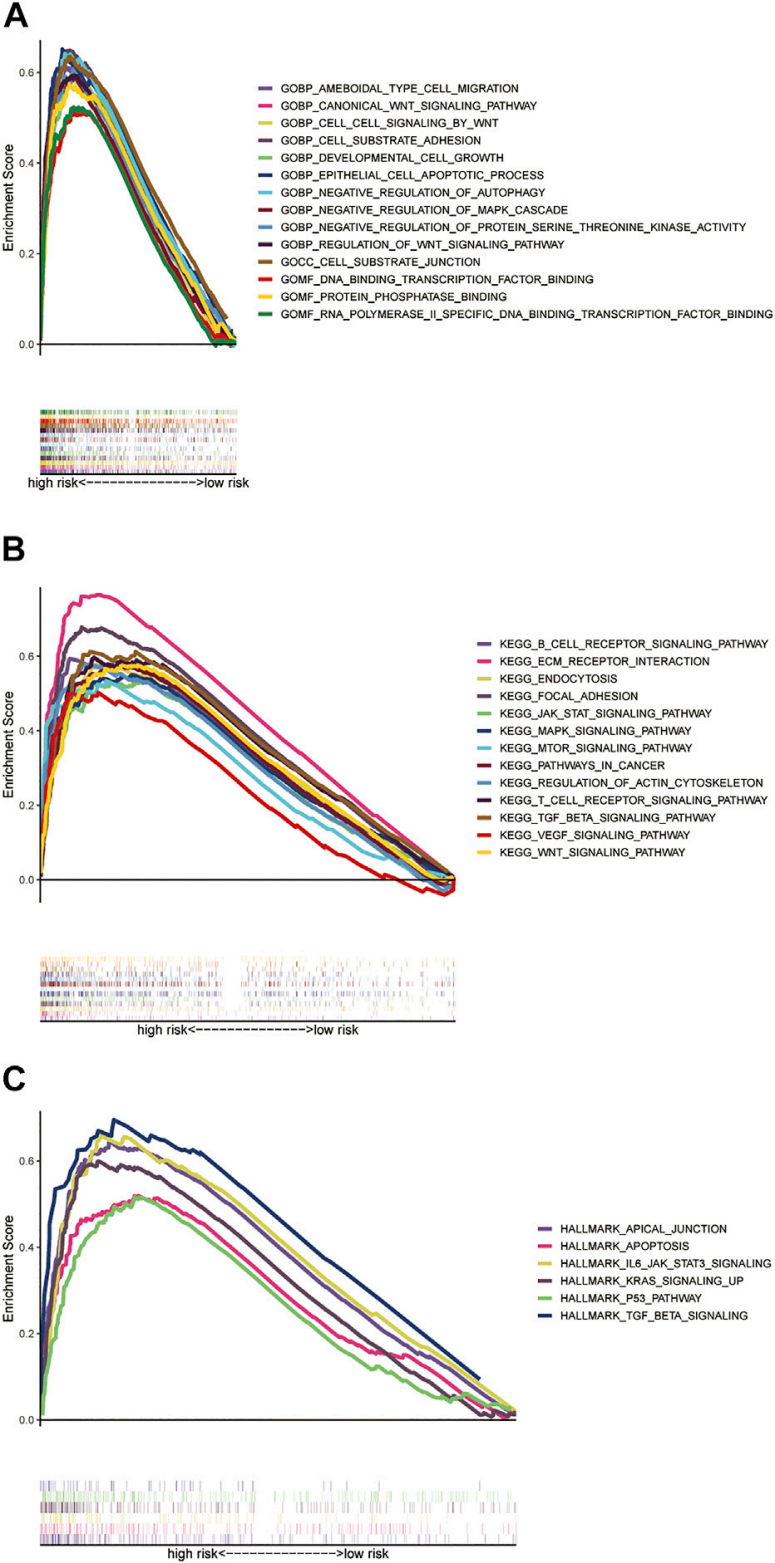
GSEA analysis of the prognostic model. **(A)** Highly related GO terms in the high-risk group. **(B)** Highly related KEGG pathways in the high-risk group. **(C)** Hallmark pathways in the high-risk group (all *p* < 0.05; FDR <0.25; |NES| > 1.5).

For the exploration of differences in biological effects between high- and low-risk groups on the basis of the risk score, we calculated the gene expression fold change between high- and low-risk groups on the basis of the risk score, and then, GSEA analysis was performed to search GO and KEGG pathways in the entire set. The highly related GO terms in the high-risk group were as follows: GOBP_CANONICAL_WNT_SIGNALING_PATHWAY and GOBP_NEGATIVE_REGULATION_OF_MAPK_CASCADE. The highly related KEGG pathways in the high-risk group were as follows: KEGG_JAK_STAT_SIGNALING_PATHWAY, KEGG_PATHWAYS_IN_CANCER, and KEGG_WNT_SIGNALING_PATHWAY. The highly related hallmark pathways in the high-risk group were HALLMARK_APOPTOSIS, HALLMARK_KRAS_SIGNALING_UP, and HALLMARK_P53_PATHWAY (*p* < 0.05 and FDR <0.25). Different color curves stand for different pathways (Figures 6A–C).

### Exploration of immunity-related factors and drug treatments

**FIGURE 7:**
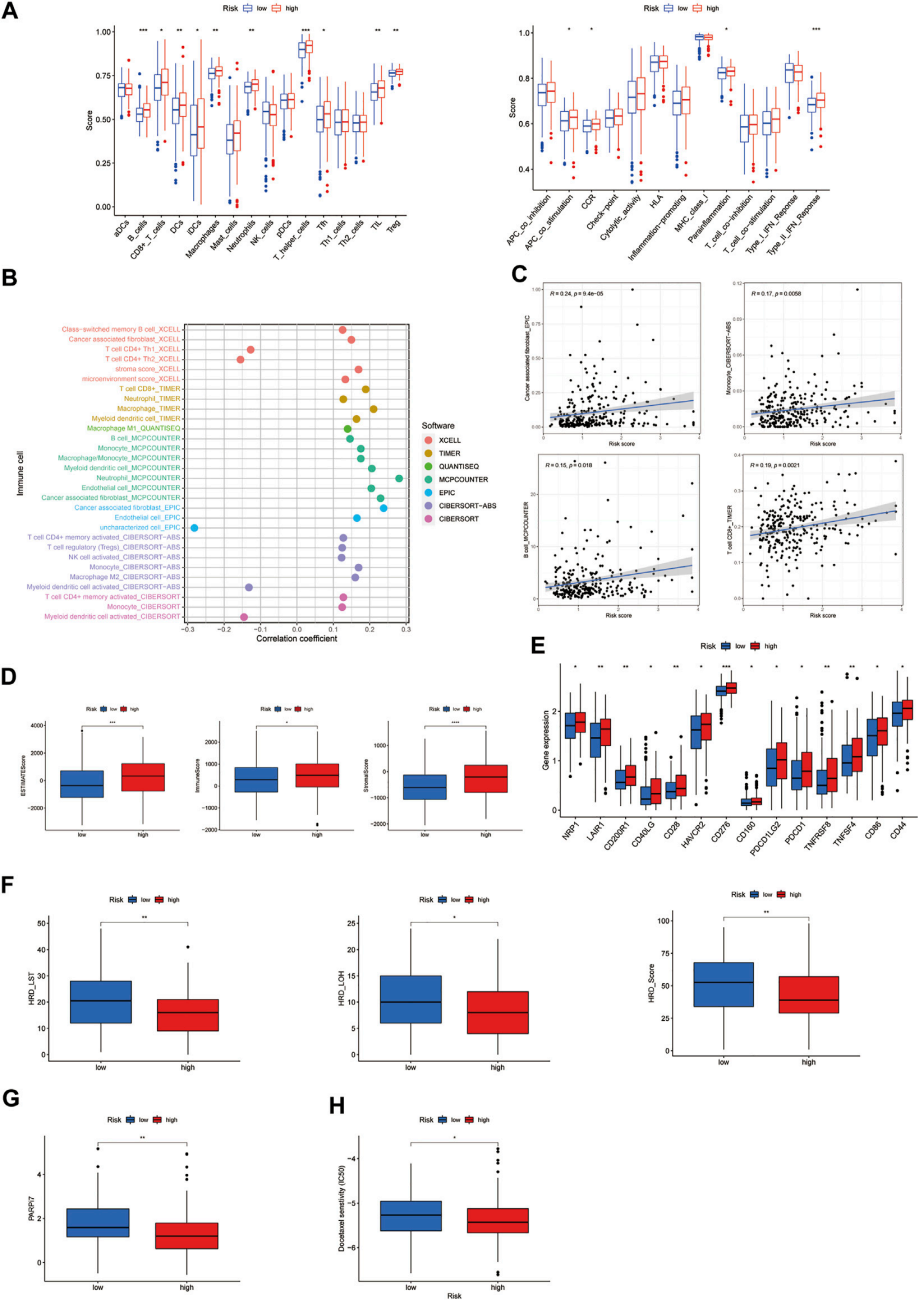
Investigation of immunity-related factors and drug treatment in the high- and low-risk subgroups. **(A)** Abundance of the immune cell in epithelial ovarian cancer (EOC). **(B)** Immune cell bubble of risk groups. **(C)** Correlation between the risk score and immune cells. **(D)** Comparison of immune-related scores between the low- and high-risk groups. **(E)** Difference in the checkpoint expression between the risk groups. **(F)** Comparison of homologous recombination deficiency (HRD) between the high- and low-risk groups. Large scale transitions, LST; loss of heterozygosity, LOH. **(G)** Comparison of 7-gene DNA-repair deficiency signature (PARPi7) between the high- and low-risk groups. **(H)** Chemotherapy sensitivity prediction in risk groups. * *p*-value < 0.05, ** *p*-value < 0.01, and *** *p*-value < 0.001.

The ssGSEA results showed there were a lot of significantly different abundances of the immune cell and immune cell functions in EOC, such as B cells, TIL, neutrophils, and type-II IFN response (Figure 7A). Multiple immune cells exhibited different expressions in high- and low-risk groups by the different platforms, such as MCPcounter, EPIC, and xCell (*p* < 0.05, Figure 7B, Supplementary Table S4). Moreover, we also discovered that the higher risk score had more association with immune cells such as B cells and T cells (Figure 7C). All of the findings demonstrated the high-risk group employed a higher immune infiltration status. Furthermore, the high-risk group owned higher ESTIMATE scores, immune scores, and stromal scores, implying different TME statuses, than the low-risk group (*p* = 0.00041, *p* = 0.033, and *p* = 3.5e-5; Figure 7D). The results of a comparative analysis of immune checkpoints of different risk scores are displayed (Figure 7E). We also found many immune checkpoints exhibited higher expression in the high-risk group, such as CD28 and CD276. Meanwhile, the large-scale transition (LST) score, loss of heterozygosity (LOH) score, HRD score, and 7-gene DNA-repair deficiency signature (PARPi7) were found to be lower in the high-risk group than those in the low-risk group (*p* = 0.0036, *p* = 0.03, *p* = 0.0071, and *p* = 0.0059; Figures 7F,G). Such significant results may provide an additional strategy for selecting patients to treat with a PARP inhibitor. We also found that PARP inhibitor drugs (olaparib and rucaparib) showed lower IC_50_ concentrations in the high-risk group (Supplementary Figures S1A,B). Furthermore, we could also find that the drug docetaxel, which has been applied to EOC therapy, showed a lower IC_50_ concentration in the high-risk group (Figure 7H).

### Three distinct expression clusters characterized by consensus clustering analysis

**FIGURE 8:**
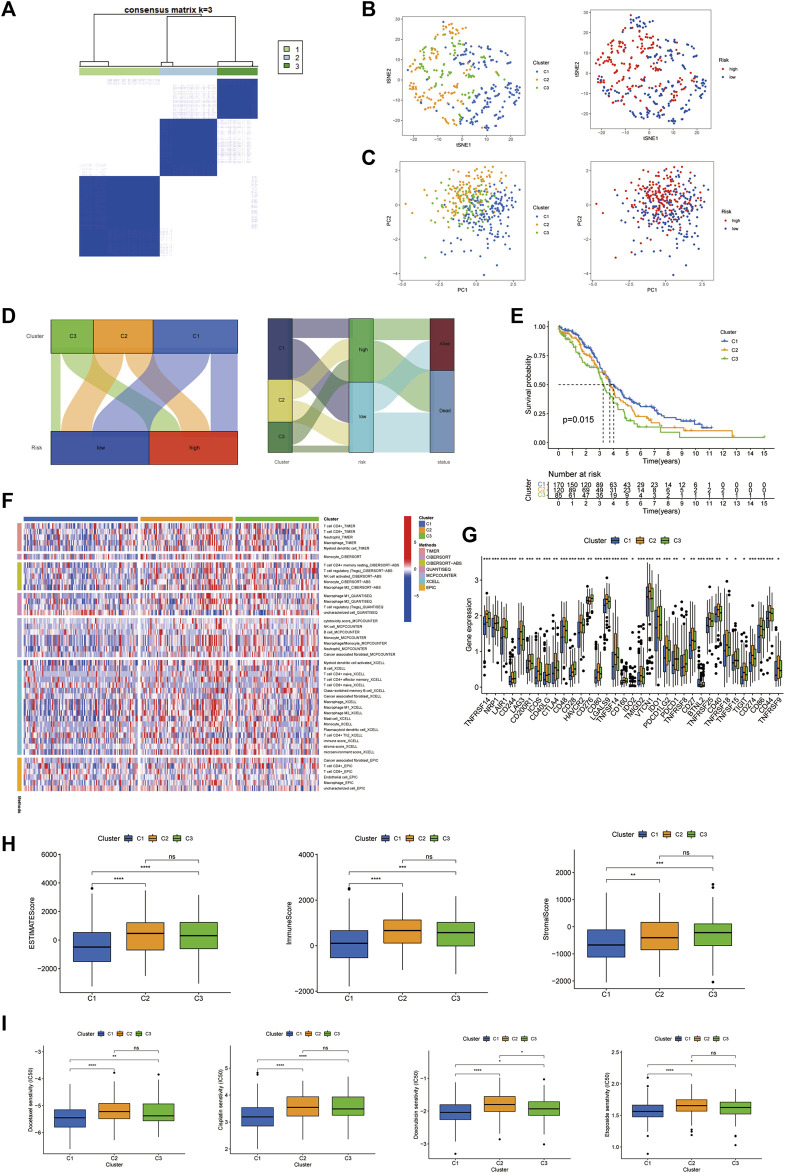
Three distinct expression clusters characterized by consensus clustering analysis. **(A)** Patients are divided into three clusters by ConsensusClusterPlus. **(B)** t-SNE of three clusters. **(C)** Principal component analysis (PCA) of risk groups and clusters. **(D)** Sankey diagram of clusters with their risk types and survival status. **(E)** Kaplan−Meier survival curves of overall survival (OS) in three clusters. **(F)** Heatmap of different algorithms of immune infiltration levels in the three clusters. **(G)** Different checkpoints’ expression levels among the clusters. **(H)** Comparison of immune-related scores in clusters. **(I)** Chemotherapy drug sensitivity analysis in the three clusters.

Different gene expression clusters usually displayed different immune microenvironments, leading to different immunotherapeutic strategies and responses, so we conducted consensus clustering based on the five HRRlncRNA expressions which formed the risk model. The three distinct clusters displayed (Figure 8A) t-SNE and PCA analysis of five HRRlncRNA expressions, which showed three clusters, and the pre-defined high- and low-risk groups could also be clearly divided into two clusters (Figures 8B, C); the Sankey diagram was adopted to display relationships of clusters with their risk types and survival status (Figure 8D). Survival analysis displayed a significant difference between the three clusters (Figure 8E), and the heatmap of different algorithms of immune infiltration levels in the three clusters is shown in Figure 8F. Furthermore, we analyzed the checkpoints' expression levels and TME status in these clusters. We could clearly see multiple checkpoints, such as LAG3 and CD28, owned significant differences (Figure 8G). ESTIMATE scores, immune scores, and stromal scores exhibited significant level differences in clusters 1 and 2 but not in cluster 3 (Figure 8H). Chemotherapy drug analysis demonstrated that drugs, such as docetaxel, also showed significant differences in high- and low-risk groups, as shown in Figure 7H. Doxorubicin displayed significant differences in pairwise comparisons, but the other three drugs showed differences mainly in clusters 1 and 2 (Figure 8I).

## Discussion

PARP inhibitors, antiangiogenic agents, and immunotherapy are the most promising targeted therapies for EOC in recent years. In the time of precision medicine, it is important to select the appropriate patients to benefit from the targeted therapy. Further understanding of the molecular characteristics associated with HRD could lead to an introduction of broader patients benefiting from PARP inhibitors. To the best of our knowledge, our study is the first systematic and comprehensive analysis of HR-associated lncRNAs in EOC. A risk model was identified based on the five HRRlncRNAs, namely, AC138904.1, AP001001.1, AL603832.1, AC138932.1, and AC040169.1. Furthermore, we divided the entire set into three clusters based on the five HRRlncRNAs, which may provide more data support for the stratification of patients, such as clinical outcomes, chemotherapy sensitivity, response to PARP inhibitors, and immunotherapy.

In this study, we first integrated the gene profiles of TCGA and GTEx, combined with HRRGs from previous research studies, and obtained 206 differentially expressed HRRlncRNAs through co-expression and differential expression analysis. Univariate Cox regression, LASSO regression, and multivariate Cox regression analyses were further performed. Finally, a five-HRRlncRNA model was determined, which could divide EOC patients into high-risk and low-risk groups and with an obvious difference in survival time between the two groups. We validated the risk model in the training set, testing set, and entire set *via* some analyses such as Kaplan−Meier analysis, ROC analysis, DCA, and IC_50_ prediction, and the results showed that the five-HRRlncRNA model had a good predictive ability. More importantly, the low-risk group employed higher HRD_LST, HRD_LOH, and HRD scores. Cancers with the HRD phenotype usually exhibit a high response to chemotherapy, especially platinum compounds in different treatment lines (Pennington et al., 2014). As for PARP inhibitors, they might be more suitable for patients with HRD caused by different etiologies. The degree of HRD strongly correlates with LOH, telomeric allelic imbalance (TAI), and the number of LSTs in the chromosomes (Timms et al., 2014). Each of these tests has its own advantages and disadvantages and could be used in a complementary manner. Currently, the best strategy to define the HRD status still needs to be determined. The risk model conducted in this study may act as a supplement role to evaluate HRD better.

Furthermore, many immune checkpoints also exhibited higher expression in the high-risk group, such as CD28 and CD276. The high-risk group also owned a higher ESTIMATEScore, ImmuneScore, and StromalScore, implying different TME statuses, than the low-risk group. It is well-known that different gene expression clusters usually displayed different immune microenvironments, leading to different immunotherapeutic strategies and responses (Deberardinis, 2020). Thus, to deeply discuss the interior relation between the five HRRlncRNAs and TME, we divided patients into three clusters in EOC based on the expression of the five HRRlncRNAs. The multiple checkpoints, such as LAG3 and CD28, presented a significant difference among the three clusters. LAG3 is the third cancer immunotherapy to be targeted in the clinic, consequently garnering considerable interest (Andrews et al., 2017). The CD28 costimulatory pathway is a vital pathway that can signal the activation of naïve T cells (Esensten et al., 2016). In addition, there was a higher immune score and higher activity of the immune checkpoint genes and chemotherapy sensitivity in cluster 2 compared with cluster 1. The results may help identify patients who would benefit from the immune checkpoint inhibitors in EOC.

As for the five HRRlncRNAs in the risk model, the research with regard to their role in tumors is just beginning. A recent report has pointed out that AC138904.1, combined with the other seven ferroptosis and iron metabolism-related lncRNAs, could independently predict survival and therapeutic effects in ovarian cancer patients (Feng et al., 2022). AP001001.1 was a novel m6A-related lncRNA pair signature for predicting the prognosis of gastric cancer patients (Wang et al., 2022). AL603832.1, as an m6A RNA methylation-related lncRNA, might serve as crucial mediators of the tumor microenvironment of head and neck squamous cell carcinoma (Feng et al., 2021). However, the roles of AC138932.1 and AC040169.1 have not been studied in tumors up to date. Our study may provide more enriched evidence of the role of lncRNAs in tumors, especially in EOC. It still needs further study.

However, there are still some limitations to this study. First, our risk model was only validated internally and not further validated with other external data. Second, it is necessary to further study the possible functions and mechanisms of these five HRRlncRNAs in combination with laboratory experiments. In addition, a larger sample size is needed to verify the accuracy of the risk model and molecular subtypes in the future.

## Conclusion

All the aforementioned results suggested that our risk model based on five HRRlncRNAs may be a potential index for evaluating the clinical outcomes, response to PARP inhibitors, and immunotherapy in patients with EOC. Moreover, we also divided EOC patients into three clusters according to the expression of the five HRRlncRNAs. Different clusters presented the significant difference in the TME status, which may aid to screen patient candidates for immunotherapy in the future.

## Data Availability

The original contributions presented in the study are included in the article/Supplementary Material; further inquiries can be directed to the corresponding author.

## Supplementary material

SUPPLEMENTARY TABLE S1
Network data on homologous recombination (HR)-related genes and lncRNAs.

SUPPLEMENTARY TABLE S2
All differentially expressed homologous recombination (HR)-related lncRNAs (HRRlncRNAs) in epithelial ovarian cancer (EOC).

SUPPLEMENTARY TABLE S3
Data on 29 homologous recombination (HR)-related lncRNAs (HRRlncRNAs) significantly correlated with overall survival (OS) of epithelial ovarian cancer (EOC) patients.

SUPPLEMENTARY TABLE S4
Profile of significantly different infiltrated immune cells between risk groups in different platforms.

SUPPLEMENTARY FIGURE S1
PARP inhibitor sensitivity prediction in risk groups. * *p*-value < 0.05, ** *p*-value < 0.01, and *** *p*-value < 0.001.
