# Supplementary figures and images for: Deciphering the expression patterns of homologous recombination-related lncRNAs identifies new molecular subtypes and emerging therapeutic opportunities in epithelial ovarian cancer
Source: Front Genet. 2022 Sep 29;13:901424. doi: 10.3389/fgene.2022.901424 (PMC9557066; doi:10.3389/fgene.2022.901424)

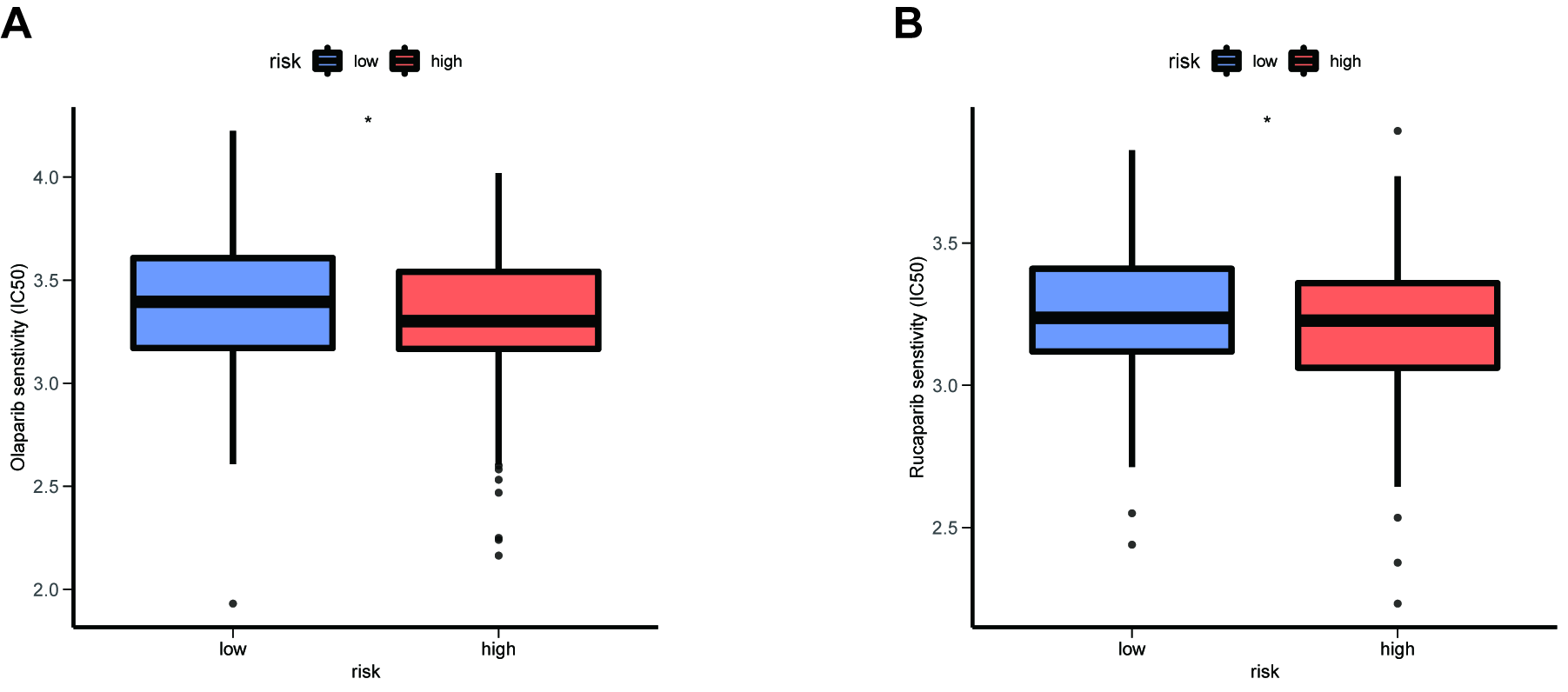

Supplement: Supplementary file 5 [file Image1.TIF]
